# Genetics and management of locally advanced carcinomas of the head and neck: role of altered fractionation radiotherapy

**DOI:** 10.4155/fsoa-2018-0058

**Published:** 2018-10-26

**Authors:** Francesco Perri, Franco Ionna, Paolo Muto, Massimiliano Di Marzo, Francesco Caponigro, Francesco Longo, Giuseppina Della Vittoria Scarpati, Giuseppe Di Lorenzo, Mario Giuliano, Raffaele Solla

**Affiliations:** 1 Head & Neck, Soft Tissue Sarcoma Medical Oncology Department, National Tumor Institute of Naples, IRCCS G Pascale, Napoli, Naples, Italy; 2 Otolaryngology Unit, Department of Head & Neck, Soft Tissue Sarcoma & Thyroid Cancer, National Tumor Institute of Naples, IRCCS G Pascale, Napoli, Naples, Italy; 3 Radiation Therapy Department, National Tumor Institute of Naples, IRCCS G Pascale, Napoli, Naples, Italy; 4 Head & Neck – Soft Tissue Sarcoma Unit, IRCCS G Pascale, Naples, Italy; 5 Medical Oncology Unit, POC SS Annunziata, Taranto, Italy; 6 Department of Clinical Medicine & Surgery, University Federico II, Naples, Italy; 7 Department of Medical Oncology, Lester & Sue Smith Breast Cancer Center, Baylor College of Medicine, Houston, TX, USA; 8 Department of Radiation Oncology, Italian National Research Council, Institute of Biostructure & Bioimaging, Naples, Italy

**Keywords:** accelerated, cyclin D1, EGFR, genetics, head and neck, HPV, hyperfractionated, P16, radiation therapy, radiotherapy, translational research

## Abstract

Squamous cell carcinoma of the head and neck (SCCHN) accounts for 5–7% of all malignancies. About 60% of newly diagnosed SCCHN are detected as locally advanced disease. Chemoradiation is a standard option and response rate to it is variable. Recently, a genetic classification of SCCHN has been proposed by Chung *et al*., who categorized all SCCHN into four subtypes. The basal-like variant is characterized by high expression of EGFR. Literature data suggest higher efficacy of accelerated and/or hyperfractionated radiotherapy, if compared with conventional radiotherapy in the subgroup of patients with high EGFR expression. In this review, we will describe the genetic factors able to guide treatment choice, with a focus on EGFR expression.

## Lay abstract

Management of head and neck squamous cell carcinomas is very challenging, with malignancies often strongly dampening the quality of life of the patients. Surgery has gradually lost its importance and it is often replaced by combined chemoradiotherapy. Response to chemoradiotherapy is very variable and, at present, it is not possible to know which patients will benefit from it. Genetic assessment of head and neck squamous cell carcinomas may help clinicians to perform a well-shaped therapy, adapted on the basis of the patient's genetic features. This strategy is presently experimental, but it may become the ‘standard’ in the future.

## The role of radiotherapy in locally advanced squamous cell carcinoma of the head & neck

Radiation therapy is often chosen as primary treatment for locally advanced squamous cell carcinoma of the head and neck (LA-SCCHN), mainly due to their unresectability. Moreover, upfront chemoradiation or radiotherapy alone (for unfit patients) represents the preferred options also for some subgroups of SCCHN, including hypopharyngeal, oropharyngeal and supraglottic laryngeal primitives. In the last decade, radiation therapy has become a very important treatment for SCCHN [1–4], since over the years many new techniques able to conform the maximum dose to the target have been developed. 2D treatments have slowly been replaced by conformal-3D techniques and, in turn, the latter have almost been substituted by intensity-modulated radiation therapy.

Today, intensity-modulated radiation therapy and volumetric-modulated arc therapy techniques are commonly employed with or without systemic therapy in patients with LA-SCCHN, achieving good overall response rates (ORR), ranging from 75 to 90% [5–10].

Nevertheless, tumor responses to these techniques vary widely, and currently it is not possible to establish which patients will particularly benefit from radiation or chemoradiation. Of note, improving radiotherapy techniques does not always lead to a better prognosis and the possible explanation of this phenomenon lies in the genetics of SCCHN.

## Genetics of SCCHN

SCCHN are a very heterogeneous group of disease, for both clinical and molecular aspects. In 2004, Chung *et al*. identified four distinct subtypes of SCCHN, namely basal-like, classical, mesenchymal and atypical variants, by microarray analysis of 582 genes. The so called ‘basal-like variant’ displayed high EGFR expression at immune-staining, associated to a well-differentiated (keratinizing) histology. The ‘mesenchymal’ subtype showed overexpression of genes typical of fibroblasts, including vimentin and syndecan, suggesting the activation of the epithelial–mesenchymal transition process in the cancer cells. The ‘classical’ variant showed high level of antioxidant enzymes, such as glutathione-S-transferase and thioredoxin reductase, which are genes highly expressed in the epithelium of strong smokers. Finally, the ‘atypical’ variant did not display keratinizing histology and appeared to be poorly differentiated and to express low level of EGFR [11]. This line of research has been recently resumed by Walter *et al*. who performed a similar analysis in patients affected by SCCHN, but, in addition, they evaluated the expression of other crucial genes, such as CCND1, TP53 and PIK3CA, and, mainly, they also assessed the status of human papillomavirus (HPV). As results, they were able to further divide the so called ‘atypical subtype’ into two classes, namely HPV related and not HPV related [12].

Summing up, the final result of these researches (Chung and Walter) was the discovery of several entities that differ widely among themselves. Prognosis was very different in these subtypes, as it was worse in the ‘classical’ and ‘basal-like’ variants.

We can summarize the above-described results highlighting that SCCHN are not a single entity, but they should be divided on the basis of their genetics. As an example, HPV-related SCCHN, which belongs to the ‘atypical’ variant described by Chung, shows typical features, such as low number of gene mutations, low EGFR expression and a wild-type status for TP53 and CyclinD1. Clinically, HPV-related tumors are characterized by good chemo- and radio-sensitivity [13,14]. On the other hand, alcohol and tobacco-related SCCHN (basal like, classical, mesenchymal and part of atypical) are more likely to be chemo- and radio-resistant; they often harbor high number of mutations, among which TPF5 and CCND1 (the gene encoding for Cyclin D1) are particularly interesting [15–18].

Basal-like variants of SCCHN are often HPV negative and are frequently related to alcohol and tobacco consumption. Interestingly, the basal-like variant is characterized by high EGFR expression, which often corresponds to EGFR gene amplification. This genome aberration has been shown to be mutually exclusive with HPV status in several studies [19–21].

In conclusion, of the above-described features is that SCCHN showing high EGFR expression are almost always not virus-related entities and, described as basal-like variant by Chung, often shows EGFR gene amplification and strong correlation with alcohol and tobacco consumption. Basal-like SCCHN variant, shows also poor prognosis in clinical trials.

## Genetics of SCCHN & implications on therapy

Given the high chemo- and radio-sensitivity of HPV-related SCCHN, locally advanced tumors may be treated with conservative strategies. Recently, Cmelack *et al*. have presented data of a Phase II study in which HPV-related SCCHN were treated with induction chemotherapy docetaxel, cisplatin and 5-fluorouracil (TPF) followed by a de-escalated chemoradiation regimen consisting of a total of 50 Gy associated with the anti-EGFR monoclonal antibody cetuximab. Preliminary results showed that these patients had better outcome compared with those with HPV-negative tumors who were treated with standard (70 Gy) radiotherapy [22]. In the future, the acquisition that some SCCHN are virus-related diseases can be sufficient to manage them with conservative and underpowered chemoradiotherapy, thus avoiding demolitive surgery and positively impacting not only on survival but also on quality of life of the affected patients. Nevertheless, it is important to clarify that not all HPV-positive SCCHN are also HPV-related malignancies, because the HPV is not always the ‘main driver’ of cancerogenesis. So, our efforts must be aimed at clearly dividing virus-related tumors from nonvirus-related ones, and this can be achieved by choosing the most suitable markers typical of related virus tumors (such as p16 overexpression, *TP53* wild-type status and Cyclin D1 wild-type status).

On the other hand, smoke and tobacco-related SCCHN seem to be chemo- and radio-resistant, thus conservative treatments might be discouraged in these categories of patients.

However, a subgroup of HPV nonrelated SCCHN, in particular those belonging to the ‘basal-like’ variant, could be treated with radiation or chemoradiation, albeit they are considered radio resistant. The reason for this statement lies in the particular biological features of these tumors.

## EGFR & response to radiation therapy

EGFR is a tyrosine kinase growth factor receptor, and a member of the HER family. EGFR activation triggers a phosphorylation cascade mediated by the PI3K–PTEN–AKT, MAPK, ERK and Jak/STAT pathways, and ultimately promotes proliferation, invasion, angiogenesis, and metastatic spread [23–25]. Aberrant activation of EGFR signaling in SCCHN can be mediated by several mechanisms, including EGFR gene amplification, overexpression of EGFR and its ligands, establishment of autocrine/paracrine loops, EGFR mutation/polymorphism and transactivation by other receptor tyrosine kinases [26,27]. Importantly, EGFR overexpression correlates with poor prognosis in SCCHN, independently from the molecular mechanism of EGFR activation. The main reason explaining this phenomenon may be the accelerated tumor cell repopulation after radiation therapy. Indeed, marked EGFR overexpression leads to constitutive activation of the downstream pathway effectors, with generation of sustained proliferative ligand-mediated signals. Evidence from clinical trials demonstrated a positive correlation between the intensity of EGFR expression and the proliferation index Ki-67 in SCCHN. The repopulation effect derived from proliferation increase may counteract the effects of radiotherapy [28–30].

Several clinical trials have clearly demonstrated that high EGFR expression strongly correlates with poor prognosis and lower response rate after standard radiotherapy or chemoradiation in patients affected by LA SCCHN [31,32].

A potential strategy to circumvent this accelerated cells repopulation may be increasing the total number of radiotherapy fractions and simultaneously reduce the delivery interval, as recently suggested in a large randomized trial [33].

Moreover, several randomized controlled studies have shown that increasing the rate of dose accumulation per week leads to an increased tumor-control probability in SCCHN [34–37].

Conventional fractionation radiotherapy is generally administered delivering a dose of 44/46 Gy in 22/23 fractions upon a large volume (including the primary tumor, any involved lymph nodes and the relevant area of lymphatic drainage), followed by an additional 22/24 Gy in 11/12 fractions upon a small volume (including the primary tumor and the known nodal involvement with a margin). Thus, a total dose of 66/70 Gy in 2-Gy fractions is delivered with one fraction per day, 5 days per week, during a planned total overall treatment time of 45 days.

On the other hand, altered fractionation radiation therapy schemes concentrate more fractions in a small time interval, increasing the radiation dose administered in the time unit. Continuous accelerated hyperfractionated radiation therapy (CHART), for example, consists in the administration of 1.5 Gy per fraction, three fractions per day, with a strict 6 h interval between each fraction. Radiotherapy is delivered over 12 consecutive days, always starting on a Monday, treating on Saturday and Sunday, and finishing on the Friday of the second week. The large volume receives 37.5 Gy in 25 fractions, and the small volume receives an additional 16.5 Gy in 11 fractions, thus delivering a total dose of 54 Gy in 36 fractions in just 12 days to the gross tumor volume.

In a Phase III randomized trial, Soren *et al*. compared a standard fractionating regimen versus CHART, in patients affected by LA SCCHN. The two regimens were able to obtain similar ORR, but a subgroup analyses revealed that in patients with high EGFR expression at immunostaining (>25% of the total cellularity in tissue samples), CHART was associated with a better response rate and survival [34].

In another Phase III trial, carried out by Bentzen *et al*., patients affected by LA SCCHN were randomized to receive an accelerated or a standard-fractionated adiation therapy (RT). As results, high EGFR expression showed a trend as independent determinant of overall survival, progression-free survival and locoregional recurrence rate (LRR) in the accelerated arm, although statistical significance was not reached. In addition, immunohistochemical analysis performed in pretreatment tumor biopsies revealed a beneficial role of high EGFR expression in patients assigned to CHART, compared with those receiving conventionally fractionated RT. Specifically, in a subgroup of patients showing high EGFR expression (cells with EGFR membrane staining ≥ 25%) a significant benefit in 3-year locoregional control rate was observed in the CHART arm compared with the conventionally fractionated RT arm. No difference between the two treatment arms was observed in the low EGFR-expressing group [35].

Eriksen *et al*. conducted a subgroup analysis on 803 patients enrolled in the DAHANCA 6 and 7 trials, treated with moderately accelerated radiotherapy or alternatively with conventional radiotherapy. Again, tumors with high EGFR expression responded better to moderately accelerated radiotherapy, compared with low EGFR-expressing carcinomas. Interestingly, tumors with high EGFR levels and well/moderate differentiation responded particularly to a moderate acceleration of treatment in terms of locoregional control, suggesting a potential role of other factors besides EGFR in influencing response to radiotherapy [36].

Suwinsky *et al*. analyzed tumor samples from 148 LA SCCHN patients treated with different radiotherapy regimens. They found that in addition to high EGFR immunohistochemical expression, high Ki-67 and absence of *TP53* mutations also significantly impacted predicted benefit from accelerated treatment, in terms of ORR [37].

Taken together, the aforementioned evidence reinforces the hypothesis that altered fractionating RT regimens, including CHART, can counteract the accelerated cell repopulation after radiotherapy in patients whose tumors express high EGFR levels.

## Conclusion

SCCHN belong to a heterogeneous group of malignancies, comprising different entities, which profoundly differ among each other with regard to biology, response to therapy and prognosis. Emerging data support the use of conservative (not surgical) treatments in HPV-related SCCHN, as they are particularly chemo- and radiosensitive [38–40]. Moreover, chemoradiation could be de-escalated in this category of patients, thus reducing the toxicity and ameliorating patients’ quality of life. There are different ways to de-intensificate the chemoradiation treatment, and the reduction of the total dose administered to the patients is the most employed in clinical trials. Nevertheless, it is possible also to modify the concomitant systemic therapy, employing a less toxic drug to be used together with radiation therapy. Several authors have compared weekly versus tri-weekly cisplatin in concomitance with radiation therapy, but the reduction of the toxicity was counteracted by the inferiority of the weekly schedule in terms of survival [41–43]. Interestingly, Chera BS *et al*. have carried out a systematic meta-analysis upon patients treated with weekly versus tri-weekly cisplatin concurrent with radiotherapy, and they demonstrated that, for patients with favorable-risk human papillomavirus-associated oropharyngeal squamous cell carcinoma, a substantially decreased intensity of therapy with 60 grays of intensity-modulated radiotherapy and weekly low-dose cisplatin produced better preservation of quality of life compared with standard therapies while maintaining excellent 3-year tumor control and survival [44].

Among nonvirus-related SCCHN, which are often smoke- and alcohol-derived, we can find the so-called basal-like variant, characterized by high EGFR expression and well differentiation grade [11,12,15].

These tumors can show resistance to radiotherapy, especially if a conventional fractionation regimen is employed. Translational studies have highlighted the fairly good responsiveness of this type of SCCHN to altered fractionating radiotherapy regimens, such as CHART [34–37]. In addition, high EGFR expression sustains tumor cell repopulation between radiotherapy fractions, as a consequence of the activation of several downstream intracellular effectors mediating cell proliferation and angiogenesis. Basing on these findings, reducing the interval between radiotherapy fractions may counteract the effect of EGFR pathway deregulation. In this regard, CHART (or other altered fractionating radiotherapy regimens) is a promising and relatively inexpensive treatment strategy, which could be employed more extensively in clinical practice with the only concern of acute radiation-related toxicities, identified in clinical trials. The only objection we could raise is the difficulty in coupling altered fractionating RT schemes with chemotherapy. We well know that chemoradiation represents the standard treatment in LA-SCCHN, being able to improve both ORR and survival, but cisplatin is very hard to administer in concomitance with CHART, due to their potential cumulative acute toxicity. A step forward may be to best incorporate altered fractionation RT schemes with systemic therapy (which may not be cisplatin, but also other less toxic agents).

Considering the clinical and molecular heterogeneity of SCCHN, we can envision that, in a near future, the treatment of patients affected by LA SCCHN should take into account their genetics, and not just focus on improving the radiotherapy techniques, which does not always lead to survival advantages.

## Future perspective

In the near future, the aim of research should be to perform a well-shaped therapy on the basis of the genetic signature of the tumor and the genetic characteristics of the patient. Specific genic mutations may confer to the tumor more aggressiveness but also more sensitivity to specific therapies. On the other hand, a number of somatic genic polymorphisms (genes encoding for DNA repair enzymes for example) found in some patients, should render them more suitable for radiotherapy and/or chemotherapy using alkylating agents. The future effort of translational research should be to study the patients and tumor characteristics, and to choose a personalized therapy.

**Executive summary**

**The role of radiotherapy in locally advanced squamous cell carcinoma of the head and neck**

- This review analyzes the importance of genetic assessment of SCCHN before performing any type of treatment, in particular, the role of altered fractionation radiotherapy regimens is described.

**Genetics of SCCHN**

- SCCHN are very heterogeneous, and we will analyze their differencies from a genetic point of view.

**Genetics of SCCHN and implications on therapy**

- Genetic differences also imply different therapeutic strategies – we will describe them based on literature data.

**EGFR and response to radiation therapy**

- EGFR expression may strongly impact on response to some types of therapy, in particular, altered fractionation radiation therapy.

**Conclusion**

- The pre-therapy assessment is crucial before performing any kind of therapeutic strategy.

**Future perspective**

- To assess a specific therapy based on the specific tumor genetic signature.
